# Investigation of the Gate Degradation Induced by Forward Gate Voltage Stress in p-GaN Gate High Electron Mobility Transistors

**DOI:** 10.3390/mi14050977

**Published:** 2023-04-29

**Authors:** Myeongsu Chae, Hyungtak Kim

**Affiliations:** Department of Electronic and Electrical Engineering, Hongik University, Seoul 04066, Republic of Korea

**Keywords:** AlGaN/GaN HEMTs, Schottky p-GaN gate, degradation, V_TH_ instability, forward gate voltage stress, two-terminal currents

## Abstract

In this work, we investigated the degradation of the p-GaN gate stack induced by the forward gate voltage stress in normally off AlGaN/GaN high electron mobility transistors (HEMTs) with Schottky-type p-GaN gate. The gate stack degradations of p-GaN gate HEMTs were investigated by performing the gate step voltage stress and the gate constant voltage stress measurements. In the gate step voltage stress test, the positive and negative shifts of threshold voltage (V_TH_) depended on the range of the gate stress voltage (V_G.stress_) at room temperature. However, the positive shift of V_TH_ in the small gate stress voltage was not observed at 75 and 100 °C and the negative shift of V_TH_ was started from a lower gate voltage at a high temperature compared to room temperature. In the gate constant voltage stress test, the gate leakage current increased with three steps in the off-state current characteristics as the degradation progressed. To investigate the detailed breakdown mechanism, we measured the two terminal currents (I_GD_ and I_GS_) before and after the stress test. The difference between the gate–source current and the gate–drain current in the reverse gate bias indicated that the increase of the leakage current was attributed to the degradation between the gate and the source while the drain side was not affected.

## 1. Introduction

AlGaN/GaN high electron mobility transistors (HEMTs) are suitable for high-frequency (RF) and high-power applications because GaN has a wide band gap of 3.2 eV, high mobility of 2000 cm^2^/V·s, high critical electric field of 3.3 MV/cm and high-temperature tolerance compared to silicon [1,2,3,4]. In addition, the two-dimensional electron gas (2DEG) channel is formed without gate voltage by spontaneous and piezoelectric polarization on the AlGaN/GaN heterojunction [5]. However, conventional AlGaN/GaN HEMTs have disadvantages, such as stability, and power consumption issues, in the field of high-power because AlGaN/GaN HEMTs operate in a normally on mode. The normally off operation is required in the field of high power to eliminate these problems and to secure the reliability of the devices. Several approaches have been employed to enable the normally off operation, such as MIS HEMTs with the AlGaN barrier recessed gate [6], the thin AlGaN barrier [7], fluorine-based plasma treatment [8], and p-type GaN gate AlGaN/GaN HEMTs [9]. Among them, the most promising method is p-GaN gate HEMTs with the p-type GaN layer grown on the AlGaN/GaN heterostructure. The conduction band edge is lifted by the formation of a p-n junction between the p-GaN and the n-AlGaN layer, resulting in the depletion of the 2DEG channel, thereby the normally off operation of AlGaN/GaN HEMTs. P-GaN gate HEMTs have been commercialized in recent years [10] and are expected to replace Si-based power devices in the future. Either Schottky contact [11,12] or ohmic contact [12,13] can be used for the gate electrode in p-GaN gate HEMTs. P-GaN gate HEMTs with ohmic-type gate contact have a small gate swing due to the issue of the gate leakage current in the forward gate bias compared to those with Schottky-type gate contact [12]. P-GaN gate HEMTs with Schottky-type gate contact can significantly reduce the gate leakage current in the forward gate bias by the reverse-biased Schottky junction diode which enables a large gate swing. However, the high electric field of the reverse-biased Schottky junction induced by positive gate voltage can cause gate reliability issues, such as time-dependent gate breakdown (TDGB) [11,14] and V_TH_ instability [15,16,17,18] in p-GaN gate HEMTs. Understanding the gate degradation mechanism in Schottky p-GaN gate HEMTs is crucial for developing reliable and high-performance p-GaN gate HEMTs. In p-GaN gate HEMTs with Schottky gate contact, the p-GaN gate stack consists of back-to-back diodes, the Schottky junction diode of the gate metal/p-GaN junction and the p-i-n junction diode of the p-GaN/AlGaN/GaN junction [14]. The p-i-n junction diode can block the gate leakage current for the reverse gate bias and the Schottky gate metal/p-GaN junction is able to block the gate leakage current for the forward gate bias. The gate degradation mechanisms of p-GaN gate HEMTs were investigated through the gate leakage properties of these back-to-back diodes.

Various measurements have been conducted to investigate the gate degradations mechanisms in p-GaN gate HEMTs [16,17,18,19]. Tallarico et al. [16] reported the role of the aluminum ratio for the degradation of threshold voltage induced by the positive bias temperature instability (PBTI) stress test. The gate constant voltage stress test was performed in Schottky p-GaN gate HEMTs that have different aluminum contents and AlGaN barrier thicknesses. He et al. [17] investigated the frequency dependence of V_TH_ instability in commercially available p-GaN gate HEMTs by the forward gate voltage stress test under static and dynamic conditions. The shift of the threshold voltage in the dynamic stress shows an opposite trend compared to the shift of the threshold voltage in the static stress in a large gate stress voltage. Tang et al. [18] investigated the mechanism of the threshold voltage degradation in commercially available p-GaN gate AlGaN/GaN transistors by performing the dynamic stress test with various gate stress voltages. A negative shift of the threshold voltage was attributed to hole injection which is confirmed by electroluminescence (EL) emission and sequential optical pumping effect.

In this paper, we studied the gate degradation of Schottky-type p-GaN gate HEMTs by performing the gate step voltage stress measurements at both room temperature and high temperatures and the gate constant voltage stress measurements at room temperature. The voltage dependence of V_TH_ instability in response to the gate stress voltage was investigated from the gate step voltage stress test at both room temperature and high temperatures. The time-dependent gate degradation was investigated by performing the gate constant voltage stress test at room temperature. To further investigate the detailed gate degradation mechanism, two terminal currents, gate–drain current (I_GD_) with source floated, and gate–source current (I_GS_) with drain floated were measured before and after the stress test. The difference between the gate–source current and the gate–drain current in the reverse gate bias was analyzed to investigate the degradation mechanism of the p-GaN gate stack.

## 2. Results and Discussion

### 2.1. Gate Step Voltage Stress

**Figure 1:**
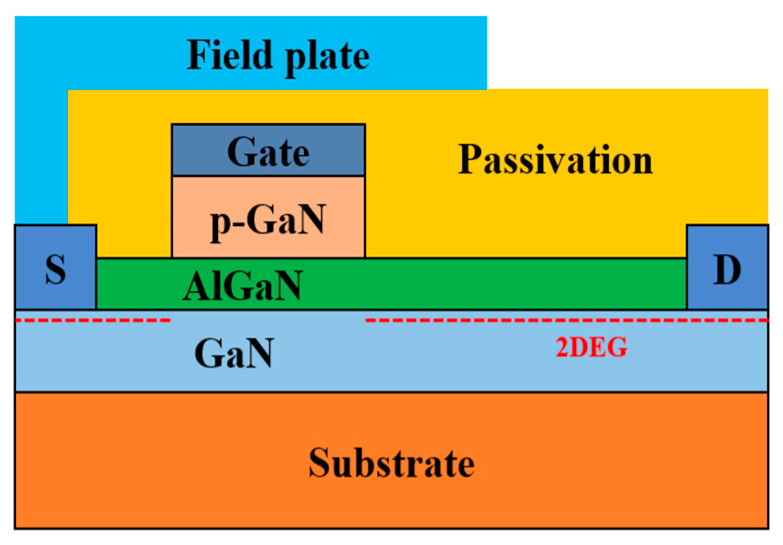
Typical device schematic of normally off AlGaN/GaN HEMT with p-GaN gate.

**Figure 2:**
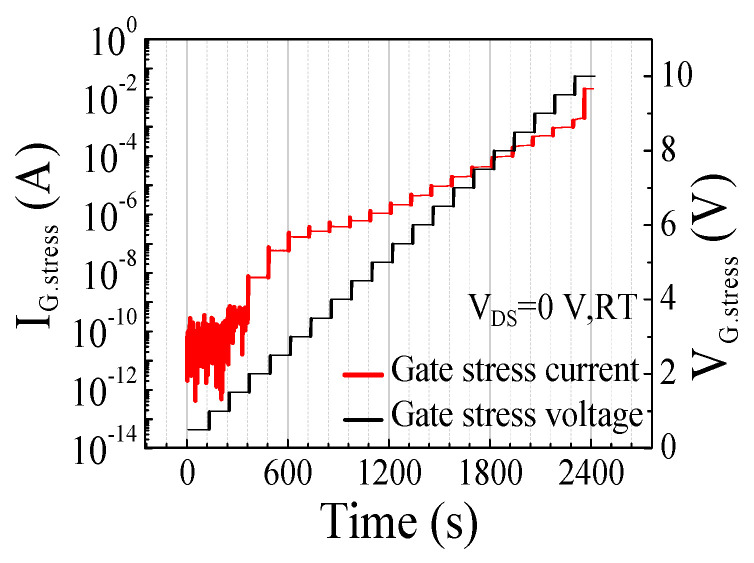
Gate step voltage stress measurement at room temperature.

The devices used in this work are commercially available 650 V p-GaN gate AlGaN/GaN HEMTs (GS-065-004-1-L) from GaN systems corporation [19]. Figure 1 shows the typical device schematic of normally off AlGaN/GaN HEMT with p-GaN gate [20]. As shown in Figure 1, the device used the p-GaN layer beneath the gate region to enable the normally off operation. The source field plate is usually employed to improve the dynamic R_on_ performance and reduce the electric field in p-GaN gate HEMTs. The gate step voltage stress measurements were performed by applying the gate stress voltage (V_G.stress_) using Keithley’s 2410 and 2651A source meters. The gate stress voltage was increased in steps of 0.5 V up to 10 V with a grounded source and drain as shown in Figure 2. Each stress step lasted for 120 s, and transfer characteristics were measured with V_DS_ = 0.5 V and a grounded source after each 120 sec–stress step to analyze the degradation of the devices. The threshold voltage was extracted using the constant current extraction method at I_D_ = 1 mA in the transfer characteristics. The gate stress current (I_G.stress_) was monitored during the stress test and increased significantly at V_G.stress_ = 10 V at room temperature, indicating a failure of the p-GaN gate stack in p-GaN gate HEMTs. The drastic increase in the gate stress current is attributed to the degradation of the Schottky junction diode in the gate metal/p-GaN junction. When the forward gate bias is applied, the high electric field of the reverse-biased gate metal/p-GaN junction can cause the degradation of the p-GaN gate stack in p-GaN gate HEMTs.

**Figure 3:**
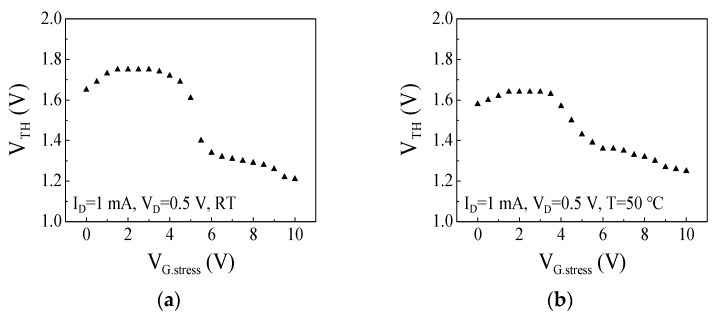

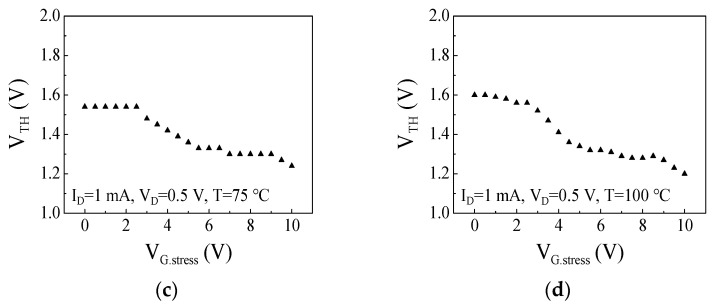
The threshold voltage for the gate stress voltage at (**a**) room temperature, (**b**) 50 °C, (**c**) 75 °C, and (**d**) 100 °C.

**Figure 4:**
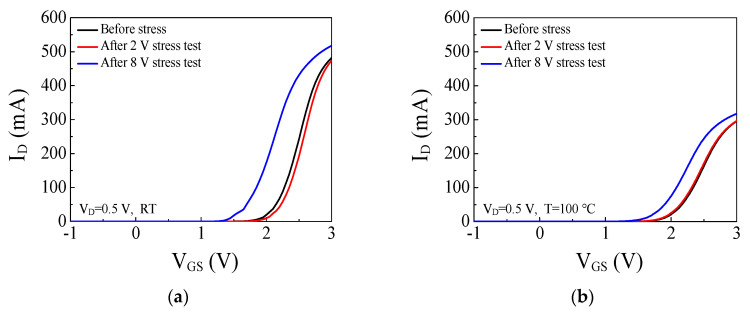
I_D_-V_G_ characteristics of the p-GaN gate HEMT before and after the gate step voltage stress test at (**a**) room temperature (**b**) 100 °C.

The gate step voltage stress tests were performed at room temperature, 50, 75, and 100 °C. Figure 3a shows a positive shift of V_TH_ in small gate stress voltages (0 < V_G.stress_ < 2 V) at room temperature which can be attributed to the trapped electrons at the AlGaN barrier and the p-GaN layer [17,21]. On the other hand, a negative shift of V_TH_ was observed in large gate stress voltages (4 < V_G.stress_ < 10 V) at room temperature which can be attributed to the trapped and accumulated holes at the p-GaN/AlGaN interface [17,21]. When the gate stress voltage increases, steeper band bending at the Schottky junction within the p-GaN can facilitate the tunneling of holes from the gate metal into the p-GaN layer. The holes injected from the gate metal compensate for the trapped electrons from the 2DEG channel. The negative shift of V_TH_ starts in the large gate stress voltage where the effect of the positive hole accumulation is greater than the effect of the negative charges by electrons. In addition, the positive shift of V_TH_ saturated at V_G.stress_ = 2 V and V_TH_ shift rarely occurred in 2 < V_G.stress_ < 4 V at room temperature. Electrons in the 2DEG channel cannot inject toward the p-GaN layer in the increased gate stress voltage due to the potential barrier of the AlGaN barrier rising by the trapped electrons at the AlGaN barrier in the small gate stress voltage. When the gate stress voltage increases, holes start to be injected from the gate metal into the p-GaN layer and accumulated and trapped at the p-GaN/AlGaN interface. The negative charges of the trapped electrons are compensated by the positive charges of the accumulated and trapped holes so there is no change in the threshold voltage in 2 < V_G.stress_ < 4 V. The positive shift of V_TH_ in the small gate stress voltage was mitigated at 50 °C compared with room temperature as shown in Figure 3b. The maximum values of the positive shift of V_TH_ are 0.11 V and 0.06 V at room temperature and 50 °C, respectively. The positive shift of V_TH_ in the small gate stress voltage was not observed at 75 and 100 °C as shown in Figure 3c,d which suggests that the trapping of electrons injected from the 2DEG channel into the AlGaN barrier and the p-GaN layer was hindered by high temperature. The trapped electrons in the AlGaN barrier and the p-GaN layer were released at high temperatures, so the positive shift of V_TH_ was not observed at 75 and 100 °C. The negative shift of V_TH_ was observed in large gate stress voltages (3 < V_G.stress_ < 10 V) at 75 and 100 °C which can be attributed to the accumulation of holes injected from the gate metal into the p-GaN/AlGaN interface, similarly as to what occurred at room temperature. The negative shift of V_TH_ was observed from a lower gate stress voltage at a higher temperature than at room temperature. The effect of the hole accumulation was likely to dominate over the effect of the electron trapping from the lower gate stress voltage at a high temperature. Unlike at room temperature, the positive charges by the accumulated holes were not compensated for by the negative charges of the trapped electrons at a high temperature. Figure 4 shows I_D_-V_GS_ characteristics of p-GaN gate HEMT in linear scale before and after the gate step voltage stress test at both room temperature and 100 °C. The drain current was lower at 100 °C compared to at room temperature as the lattice scattering of carriers occurs heavily at a high temperature. The drain current measured after the stress test with V_G.stress_ = 2 V at room temperature decreased due to the positive shift of V_TH_ as shown in Figure 4a. The drain current measured after the stress test with V_G.stress_ = 8 V at room temperature increased due to the negative shift of V_TH_. Conversely, the drain current measured after the stress test with V_G.stress_ = 2 V at 100 °C did not change due to no change in V_TH_ as shown in Figure 4b. The drain current measured after the stress test with V_G.stress_ = 8 V at 100 °C increased due to the negative shift of V_TH_ similar to room temperature.

**Figure 5:**
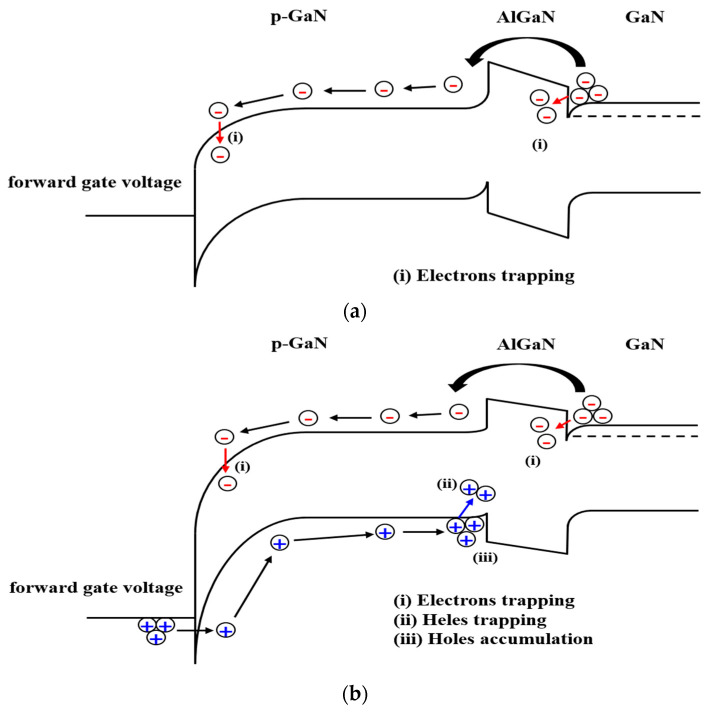
Schematic band diagrams of Schottky p-GaN gate AlGaN/GaN HEMT in (**a**) the small gate voltage and (**b**) the large gate voltage.

Figure 5 shows the energy band diagram of Schottky-type p-GaN gate AlGaN/GaN HEMT in the forward gate bias. The energy barrier of the AlGaN layer is lowered in the small gate voltage so electrons in the 2DEG channel start to spill over the AlGaN barrier and are trapped at pre-existing trap states in the AlGaN barrier and the p-GaN layer as shown in Figure 5a. The concentration of electrons in the 2DEG channel would be reduced by the trapping effect of electrons. The reduction of the concentration in the 2DEG channel causes the positive shift of V_TH_ as shown in Figure 3a. When the forward gate voltage increases, holes could inject from the gate metal into the p-GaN layer. Holes injected from the gate metal into the p-GaN layer can be accumulated and trapped at the p-GaN/AlGaN interface. The accumulation and trapping of holes injected from the gate metal occur along with the trapping of electrons injected from the 2DEG channel toward the AlGaN barrier in the large gate voltage as shown in Figure 5b. The positive charges by holes injected from the gate metal compensated for the negative charge by the trapped electrons from the 2DEG channel. The trapped and accumulated holes would increase the concentration in the 2DEG channel which could cause the negative shift of V_TH_ in the large gate voltage. He et al. [17] experimentally investigated the threshold voltage instability in p-GaN gate HEMTs by carrying out the static positive bias temperature instability (PBTI) stress measurements. Electrons in the 2DEG channel start to spill over the AlGaN barrier and some of the electrons can be captured in the pre-existing traps in the p-GaN layer and the AlGaN barrier with a relatively low gate stress voltage. The tunneling of holes from the gate metal to the p-GaN layer becomes effective at V_G.stress_ > 5 V which is dependent on the thickness of the Schottky barrier between the gate metal and the p-GaN layer. The injected holes can drift and accumulate at the p-GaN/AlGaN interface. Gu et al. [21] measured and interpreted the threshold voltage instability induced by the gate stress voltage in p-GaN gate HEMT on Si. The shift of threshold voltage and drain current degradation was observed in low gate voltages (1 < V_GS.stress_ < 2 V) which is related to the loss of carriers in the 2DEG channel. Electrons in the 2DEG channel are able to escape to the AlGaN barrier as a result of the AlGaN barrier lowering and would get trapped in the AlGaN barrier and the p-GaN layer. The reduction of the 2DEG channel could cause a positive shift of threshold voltage and drain current degradation in the low gate voltage. Holes injected from Schottky contact toward the p-GaN layer would accumulate at the p-GaN/AlGaN interface. The positive charge in the p-GaN gate stack causes an enhancement of the 2DEG channel which would result in the negative shift of the threshold voltage and the variation of the drain current.

### 2.2. Gate Constant Voltage Stress

**Figure 6:**
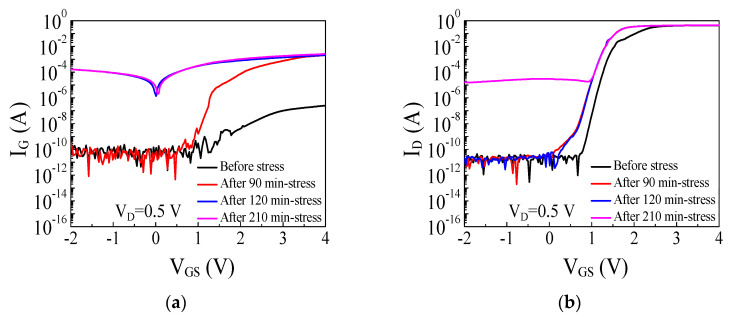
I-V characteristics of the p-GaN gate HEMT before and after the stress test with V_G.stress_ = 9.2 V: (**a**) the gate current and (**b**) the drain current.

The gate constant voltage stress test was performed with V_G.stress_ = 9.2 V for 210 min, and the transfer characteristics were measured every 30 min during the stress test to analyze the degradation of the devices. The gate stress voltage of 9.2 V was determined based on the breakdown voltage of 10 V extracted from the gate strep voltage stress test. The gate stress current was monitored during the stress test and increased significantly during the gate constant voltage stress test. The time-dependent degradations with three different stages were observed from the transfer characteristics. As shown in Figure 6a, the gate current for V_G_ > 1 V drastically increased after 90 min of the stress test, indicating that the reverse-biased gate metal/p-GaN Schottky junction failed and could not block the gate leakage current. The degradation of the Schottky junction can be attributed to avalanche breakdown induced by the impact ionization of accelerated electrons and holes [11,22]. The energy barrier of the AlGaN layer is lowered by the accumulated and trapped holes and the forward gate bias which triggers the injection of electrons from the 2DEG channel into the p-GaN layer. The electrons and holes are accelerated by the high electric field of the reverse-biased Schottky junction, and the energized carriers would cause the degradation of the p-GaN gate stack in the p-GaN gate HEMT. Wu et al. [11] studied the gate breakdown mechanism induced by the forward gate bias in Schottky p-GaN gate AlGaN/GaN HEMTs and demonstrated that the gate breakdown voltage increased in p-GaN gate HEMTs as temperature increased which is a unique result not seen in conventional AlGaN/GaN MIS HEMTs. The positive temperature dependence of the gate breakdown by the forward gate bias in p-GaN gate HEMTs can be explained by the avalanche breakdown triggered by the impact ionization. Varying degradation analyses in high-temperature environments have been studied due to the importance of temperature effect in gate constant voltage stress measurement [23]. He et al. [22] investigated the gate degradation and the physical mechanism induced by the positive gate voltage stress in Schottky-type p-GaN gate HEMTs. Electrons and holes injected into the depleted p-GaN layer can be accelerated and would induce the defect levels by bombarding the gate metal/p-GaN interface or the p-GaN layer. When a high forward gate bias is applied, electrons in the 2DEG channel can be emitted over the AlGaN barrier which was lowered by the accumulated and trapped holes at p-GaN/AlGaN interface. Electrons and holes in the depleted p-GaN region are accelerated by the high electric field of the reverse-biased Schottky junction and could cause the degradation of the p-GaN gate stack in p-GaN gate HEMTs. On the other hand, the gate current for V_G_ < 1 V maintained a low current after 90 min of the stress test, suggesting the p-i-n junction is intact and can block the gate leakage current. The gate current for V_G_ < 1 drastically increased after 120 min of the stress test, indicating that a second breakdown of the device occurred in the p-GaN gate stack. On the other hand, the drain current for V_G_ < 1 V maintained a low current even after 120 min of the stress test as shown in Figure 6b, suggesting that the channel control of the p-i-n junction is preserved and can block the leakage current. The low drain current after the stress test suggests that the second breakdown of the device was assumed to occur between the gate and the source. The drain current for V_G_ < 1 V drastically increased after 210 min of the stress test, indicating that a third breakdown occurred in the p-GaN gate stack. The third breakdown was likely to occur at the p-i-n junction or between the gate and the drain. The breakdowns of the p-GaN gate stack in p-GaN gate HEMTs occurred with three steps by the forward gate voltage stress.

**Figure 7:**
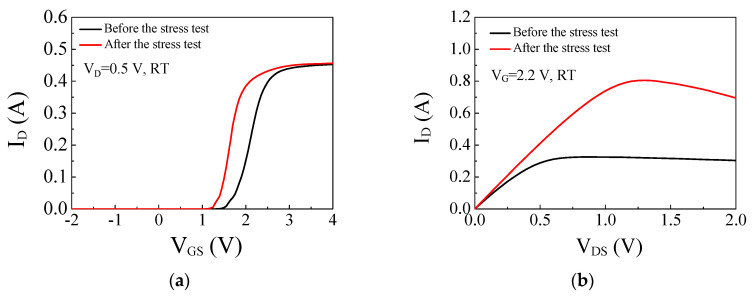
I_D_-V_GS_ (linear scale) (**a**) and I_D_-V_DS_ (**b**) characteristics before and after stress test with V_G.stress_ = 9.2 V.

Figure 7 presents I_D_-V_GS_ and I_D_-V_DS_ characteristics in linear scales and presents the effect of the Vth shift on the output current. The negative shift of V_TH_ was fairly large after the stress test, and this resulted in a significant increase of drain current measured with V_G_ = 2.2 V. The I_D_-V_D_ measurement was limited below V_G_ = 3 V due to the current compliance of the measurement system.

**Figure 8:**
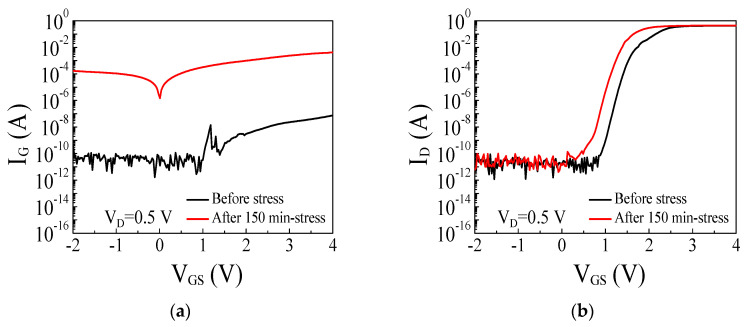
I_G_-V_GS_ (**a**) and I_D_-V_GS_ (**b**) (log scale) characteristics before and after the stress test with V_G.stress_ = 9.2 V during 150 min.

**Figure 9:**
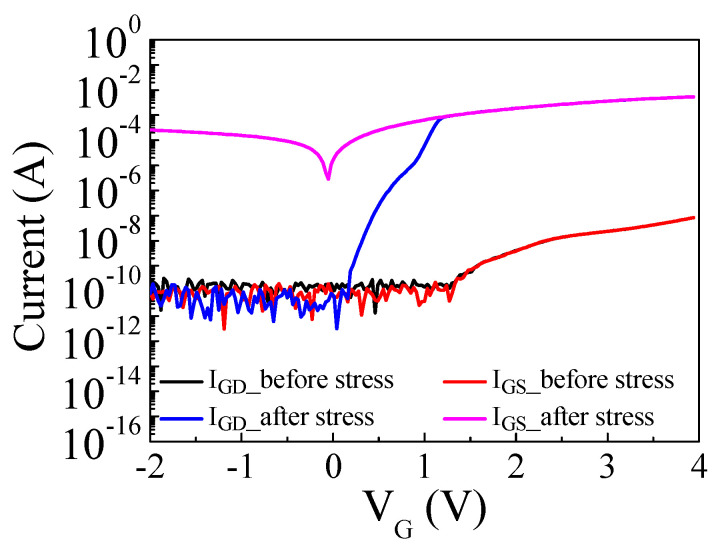
Two terminal currents before and after the stress test with V_G.stress_ = 9.2 V (I_GD_ is the gate–drain current with source floated and I_GS_ is the gate–source current with drain floated).

To gain further insights into the detailed failure mechanism of the p-GaN gate HEMTs, an additional gate constant voltage stress test was performed with V_G.stress_ = 9.2 V for 150 min, and two terminal gate currents were measured before and after the stress test, where the gate–drain current (I_GD_) was measured with source floated while the gate–source current (I_GS_) was measured with drain floated. The gate stress current was monitored during the stress test and remarkably increased, indicating the degradation of the p-GaN gate stack. The gate current drastically increased after 150 min of the stress test as shown in Figure 8a. The increase of the gate current in the forward gate bias demonstrated that the gate leakage current cannot be blocked in the forward gate bias due to the degradation of the reverse-biased Schottky junction. The drain current in the reverse gate bias maintained a low current level while the gate current in the reverse gate bias drastically increased as shown in Figure 8 which indicates that the drain side and p-i-n junction diode are intact. As shown in Figure 9, both the gate–drain current and the gate–source current in the forward gate bias drastically increased after the stress test, indicating a breakdown of the Schottky junction diode by the high electric field in the depleted p-GaN region. However, the gate–drain current in the reverse gate bias maintained the low current level even after the stress test, suggesting that the p-i-n junction is still able to block the gate leakage current and is intact. In addition, the channel control of the p-i-n junction is preserved which is confirmed by a high I_D.on_/I_D.off_ ratio of 10^10^ in the transfer characteristics as shown in Figure 8b. On the other hand, the gate–source current is approximately eight orders of magnitude higher than the gate–drain current at V_G_ = −2 V after the stress test as shown in Figure 9. The difference between the gate–drain current and the gate–source current after the stress test indicates that the degradation of the device occurred between the gate and source, while the drain side was unaffected. A leakage current path can be formed through the passivation layer between the gate and the source, between the source field plate and the gate electrode, or along the interface at the gate sidewall. Thus, the gate–source current is likely to flow through the created leakage current path, while the gate–drain current in the reverse gate bias is blocked by the p-i-n junction diode. Further microscopic studies are needed on the specific creation mechanism of the current leakage path between the gate and the source.

## 3. Conclusions

In this work, the gate degradation was observed by the forward gate voltage stress in normally off Schottky p-GaN gate HEMTs. The positive shift of V_TH_ by small gate stress voltages (0 < V_G.stress_ < 2 V) was attributed to the trapping of electrons at room temperature. The trapping and accumulation of holes by large gate stress voltages (4 < V_G.stress_ < 10 V) at room temperature resulted in the negative shift of V_TH_. However, the positive shift of V_TH_ was not observed at 75 and 100 °C because the trapped electrons were easily released at high temperatures. Additionally, the negative shift of V_TH_ started from a lower V_G.strees_ at high temperatures compared to room temperature as the accumulated holes were not compensated by the trapped electrons at a high temperature. The degradations of Schottky p-GaN gate HEMTs demonstrated three different characteristics, such as the increase of the gate current in the forward and reverse gate bias and the increase of the drain current in the reverse gate bias, during the gate constant voltage stress test. The remarkable enlargement of the drain current induced by the negative shift of V_TH_ was observed in the I_D_-V_DS_ characteristics. Two terminal gate current measurements showed only the increase of the gate–source current in the reverse gate bias. The increase of the gate–source current without the increase of the gate–drain current in the reverse gate bias demonstrated that the gate leakage’s current path between the gate and the source was created while the drain side was not affected.

## Data Availability

Not applicable.
